# Hydrogen Ion Dynamics of Cancer and a New Molecular, Biochemical and Metabolic Approach to the Etiopathogenesis and Treatment of Brain Malignancies

**DOI:** 10.3390/ijms20174278

**Published:** 2019-09-01

**Authors:** Salvador Harguindey, Julian Polo Orozco, Khalid O. Alfarouk, Jesús Devesa

**Affiliations:** 1 Institute of Clinical Biology and Metabolism, 01004 Vitoria, Spain; 2 Al-Ghad International Colleges for Applied Medical Sciences, Al-Madinah Al-Munawarah 42316, Saudi Arabia; 3 Alfarouk Biomedical Research LLC, Tampa, FL 33617, USA; 4 Scientific Direction, Foltra Medical Centre, 15886 Teo, Spain

**Keywords:** etiopathogenesis of gliomas, pH-centric anticancer paradigm, pH, NHE and proton extruders, reverting proton reversal, cellular acidification in gliomas, treatment of glioblastoma multiforme

## Abstract

The treatment of cancer has been slowly but steadily progressing during the last fifty years. Some tumors with a high mortality in the past are curable nowadays. However, there is one striking exception: glioblastoma multiforme. No real breakthrough has been hitherto achieved with this tumor with ominous prognosis and very short survival. Glioblastomas, being highly glycolytic malignancies are strongly pH-dependent and driven by the sodium hydrogen exchanger 1 (NHE1) and other proton (H^+^) transporters. Therefore, this is one of those pathologies where the lessons recently learnt from the new pH-centered anticancer paradigm may soon bring a promising change to treatment. This contribution will discuss how the pH-centric molecular, biochemical and metabolic perspective may introduce some urgently needed and integral novel treatments. Such a prospective therapeutic approach for malignant brain tumors is developed here, either to be used alone or in combination with more standard therapies.

## 1. Introduction

Our interest in the treatment of malignant gliomas (MG), mainly glioblastoma multiforme (GBM), dates from more than thirty years ago [1], and interest in the relationship of pH and cancer dates even further back to a decade earlier [2]. In order to describe the new pH-centric approach to malignant diseases, the concept of “hydrogen ion dynamics of cancer” was introduced in the early 1980s [3], a term that is increasingly used in publications in this growing field in cancer research and treatment [4].

In spite of all the efforts undertaken during the last few decades in clinical neuro-oncology and the new drugs, therapeutic methodologies and protocols employed, the prognosis of brain cancer is still dismal, with GBM showing the worst five-year survival rates among all cancers [5]. Very few patients survive more than a year, and those that do usually suffer a deteriorated quality of life, particularly in brain stem glioblastomas in children and young adults [6,7]. So far, only a few isolated cases of long-term survivors have been reported following the orthodox and traditional therapeutic approaches of maximal surgical resection, chemotherapy, immunotherapy and radiotherapy [8]. 

The cause of most brain tumors is unknown. Also, there are no environmental-associated conditions that are known to cause these malignant brain tumors, with the exception of ionizing radiation. Therefore, prevention is not a possibility. Besides, the initially promising results of intra-arterial chemotherapy have shown no real benefit in the long term, especially considering the serious and even life-threatening side effects that these procedures can induce [9]. In order to improve the regrettable therapeutic situation of MG, large multicenter studies have proposed the concerted use of several drugs through combined therapeutic protocols [10]. An interesting combination of nine different repurposed drugs have been tried recently, along with standard therapies, although they have only resulted in marginal benefits [11].

## 2. Towards a New Perspective and Clinical Approach to Malignant Gliomas 

The overall therapeutic failure in the treatment of MG makes it necessary that brand new ideas and untrodden approaches and efforts are urgently developed in order to improve the therapeutic results. The different areas of the pH-centered perspective of cancer, from etiopathogenesis to therapeutics, have been recently summarized in a recent issue dedicated to this area of research and treatment. However, MG were not considered in these publications of our group, this being the first contribution in the literature dedicated to MG [12,13]. At the same time that these conceptual changes in oncology were taking place, the interest raised by the abnormalities of the intracellular and the extracellular pH (pHi and pHe) of both normal and brain cancer cells have shown a most beneficial “side effect” in the understanding the many levels of the pH-related paradigm, and/or the hydrogen ion (H^+^) dynamics of cancer [3,13,14,15,16,17,18,19,20,21].

### 2.1. Pathological Hydrogen Ion Dynamics and Acid-Base Homeostasis in the Etiopathogenesis of Brain Tumors and Other Malignant Processes: Genetic and Epigenetic Factors

#### 2.1.1. On Etiopathogenesis

Regarding the energetics of cellular acid-base homeostasis, different proton transporters (PTs) have been implicated in the etiopathogenesis of MG. Several PTs, proton pumps (PPs) (Vacuolar-ATPases), ion channels, ion exchangers and aquaporins have been reported to be involved in the pathophysiology of brain malignancies, mainly because of their capacity to induce an intracellular alkalinization (IA), which at the same time induces multidrug resistance (MDR) [12,22,23,24,25]. Specifically, an elevated pHi mediates in the MDR resistance of MG [26], but also in other malignancies, such as cervical and ovarian cancer, to different drugs like cisplatin, vinblastine and 5-fluorouracil [27]. Contrariwise, the suppression of the activity of V-ATPase potentiates the cytotoxic effects of cisplatin by decreasing pHi [28]. 

Most importantly, cisplatin can significantly modify the intracellular pH of cancer cells inducing cytoplasmatic acidification, seemingly through a cisplatin-mediated inhibition of proton extrusion and down-regulation of NHE-1. Otherwise, the activity of NHE-1 and its effect on increasing pHi also increases cisplatin resistance to treatment [29]. As a result of these findings, both NHE and the cancer-specific pHi/pHe proton reversal can nowadays be considered to be the new and main key actors in the etiopathogenesis of MG. Indeed, the main molecular, biochemical and metabolic factor blamed for the onset of MG is the NHE exchanger isoform 1 (NHE1) and, secondarily, other NHE isoforms like NHE5 and NHE9 [30]. In summary, during the last few years, the new hydrogen ion (H^+^)-related perspective of a wide array of cancers, and of MG too, has progressed from the original Warburg approach to glycolytic cancer metabolism to a post-Warburg pH-centered paradigm. In the same line, and since the 1980s, the interest in cancer etiopathogenesis has shifted its main emphasis from intermediary metabolism and glycolysis to the selective H^+^ abnormalities of cancer cells and tissues and on its molecular relationships with H^+^-extruding transport systems and pHi/pHe regulators [31,32]. 

Thus, NHE1 has become a specific and major factor in the etiopathogenesis of MG regarding its etiology, migration, survival in hostile microenvironmental conditions and relentless progression [33,34,35]. NHE 1 un-regulation maintains an intracellular alkaline pHi, which also prevents an intracellular acidification (IAc) as the main mediator of a selective apoptosis of MG cells. Thus, an elevation of pHi also represents a key factor of multiple drug resistance (MDR) in MG, similar to other malignancies [22,23,34,36,37]. 

In the same line of cancer etiopathogenesis, the extracellular and/or microenvironmental tumoral acidification (↓pHi), induced as a consequence of the initial intracellular alkalotic deviation (↑pHi), secondarily becomes a fundamental issue of a primary importance in cancer growth. In this way, this acidic pHe becomes the ultimate mechanism to allow malignant tumors to escape from the anti-tumor immunity of the parasitized human organism. The final result is that this microenvironmental-intratumoral-extracellular (EC) low pHe situation creates a protective shield around cancer with the onset of a state of energy and immunosuppression mediated by such EC acidification-induced losses of function of T and NK cells [38,39,40,41,42].

It is worth recognizing that even in the case of a genetically-induced overexpression of NHE1 [43], genes do not seem to exert a direct influence on cellular metabolism, but they do it through the microenvironmental changes they induce. In this vein, F. Nijhout [44] integrated both ways, genetic and epigenetic, by stating: “When a compound modified by a gene is needed, it is a signal from the environment which activates the expression of the gene and never an intrinsic characteristic of the gene”. From all the late results in the field it can be concluded that NHE1 also plays a fundamental role in the local growth and activation of the metastatic process of many other malignant tumors besides MG [22,35,37,45,46,47,48]. Most significantly, the overexpression of NHE1 can be considered a widespread carcinogenic factor that is stimulated by myriad elements of different natures, all of which induce a high pHi-mediated carcinogenic response in normal cells of many different origins and irrespective of their genetic background and location (Table 1). It is most likely that at least some of these contributing factors to the etiopathogenesis of cancer can also be involved in the pathogenesis of MG through their effects on overexpressing and/or up-regulating NHE1 and the intracellular alkalinization induced by it.

**Figure 1:**
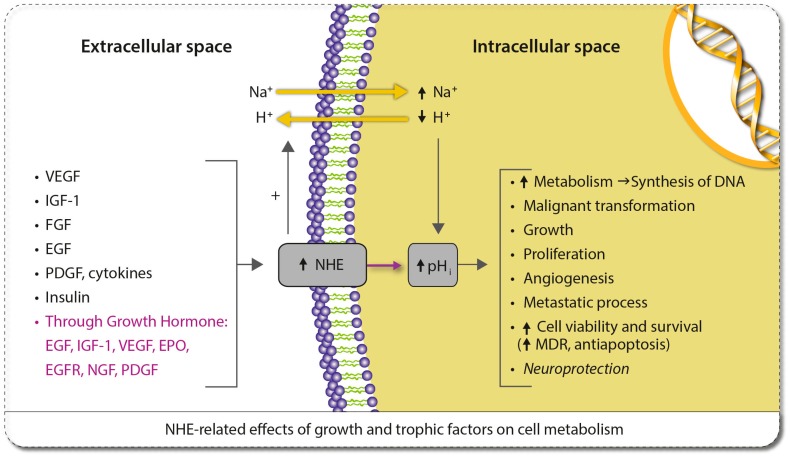
Growth and trophic factors, and cytokines that are involved in the carcinogenic expression and/or hyperactivity of NHE1 and the consequent increase in pH (modified and updated from ref [13]).

We are not aware that the possibility of a cause-effect relationship of genetic mutations of *BRCA1* and *BRCA2* with pHi and/or NHE1 expression has been made before. The intention of including *BRCA1* and *BRCA2* in Table 1 is to suggest that NHE1 and/other proton extruders, like carbonic anhydrases (CAs), can mediate in the carcinogenic action of these gene mutations in a similar, or even the same, way as happens with other gene products [44]. Table 1 also shows the many hormones, growth and trophic factors, as well as certain cytokines that over-express NHE1 and induce its pH-related pathological effects on cellular metabolism [13,21]. Human growth hormone (HGH) on its own is able to stimulate the production of a wide array of growth factors, hormones and cytokines, such as IGF-1, EGF and its receptor, VEGF, FGF, EPO, BDNF, PDGF, certain interleukins and sex steroids, some of which up-regulate NHE1 [50]. (Also see Figure 1).

During the life of Otto Warburg (Warburg died in 1970), proton extruders and all the other factors shown in Table 1 were not known. Thus, Warburg could not know that cancer cells were not acidic, as he always thought, but just the opposite [51,52]. Furthermore, we also know that Warburg was wrong in defending the theory that aerobic glycolysis was the prime cause of cancer, but it can also be said that the prime cause of cancer is the main, and perhaps universal, mediating cause of aerobic glycolysis, namely, intracellular alkalinization [13]. Indeed, this and other recent publications have also led to the conclusion that the famous Warburg Effect may be completely explained through the elevation of pHi in cancer cells [53,54,55]. 

NHE1- and pH-related pathology are receiving increased levels of attention as fundamental factors in other areas of carcinogenesis. In this vein, Hardonnière et al. have advanced a most provocative and integral explanation of human environmental carcinogenesis [56]. Even more recently, the same research group has correctly suggested that the oncogenic activity of many carcinogens of different origins and natures (Table 1) can share the same and/or similar pathways and effects on cellular H^+^ dynamics, facilitating proton gradient reversal. This opens up the possibility that the overexpression of either NHE1 and/or other proton extruders could be behind the existence of a universal mechanism responsible for the induction of environmental carcinogenesis [4]. 

The findings of these researchers, but also of other groups, suggest that the final cancer-inducing mechanisms of carcinogens like polycyclic aromatic hydrocarbons, as well as the activity of other widespread carcinogenic environmental compounds ubiquitously present in low concentrations in nature, even in groundwaters, like arsenic salts, are NHE-mediated [57]. These findings lead towards a unitarian synthesis of environmental carcinogenesis and to the conclusion that there could well exist a final and universal mediating acid-base mechanism, namely, a mechanism related to environmental H^+^ dynamics that can fully explain human carcinogenesis. Finally, Figure 1 graphically shows the growth and trophic factors whose effects are mediated by the NHE1 antiporter as H^+^ extruder, some of them also stimulating tumoral angiogenesis. For a more complete review of NHE-related proangiogenic and antiangiogenic molecules, see ref. [58].

Although in Figure 1 the human growth hormone (GH) appears as a pro-oncogenic factor, it has also been shown than this hormone can be a useful and safe treatment for many different pathologies, even in some neurodegenerative processes [13,21,59,60]. However, even in patients with a past history of neoplasia, GH replacement therapy does not appear to increase the chances of inducing a tumoral process [61,62]. Contrariwise, GH has also been considered as a “one-step” oncogene able to promote both proliferative and metastatic processes [63,64,65]. In this vein, since the existence of GH receptors in MG has been demonstrated, it seems that GH could exert actions responsible for the induction, or at least progression, of MG [66]. It can then be assumed that although systemic GH may lack a direct effect on the induction and/or progression of these tumors, some growth factors induced by GH, like IGF-1, EGF and VEGF, could negatively affect tumor growth through NHE stimulation and/or cellular alkaline pH changes, an effect that was first described from seminal publications decades ago (Figure 1) [67,68,69].

#### 2.1.2. On Treatment 

From the opposite point of view of cancer etiopathogenesis, namely, therapeutics, the pharmacological targeting and inhibition of NHE1 and other ion transporters, pumps and voltage gated sodium channels, is fundamental in inhibiting both local growth and the different stages of the metastatic process, either in MG and/or in a variety of other extracranial malignant tumors [70,71,72,73,74,75,76,77]. 

The H^+^-related perspective, as applied to the treatment of MG, has been defended by different research groups [49,78,79,80,81]. In this line, inhibiting NHE1 in MG acidifies tumor cells while normal astrocytes are not affected, a finding that open the way towards a selective and non-toxic, or minimally toxic, treatment of MG [35]. In a similar context, this therapeutic approach has been most correctly called “the Achilles heel of cancer” [82], although it also appears not to be free from some therapeutic limitations [83]. 

Furthermore, blocking Na^+^/H^+^ exchange decreases tumor growth and stimulates MG immunogenicity, besides increasing the effect of temozolomide (TMZ) [34,84]. Most surprisingly, these authors also reported that TMZ increases NHE1 protein levels in human glioblastoma cells, a feature that not only could increase TMZ resistance, but raises serious doubts about a possible deleterious effect of TMZ in the treatment of MG. On the other hand, the combination of TMZ and cariporide in a mouse glioma xenograft model significantly prolonged the survival of the mice in the same report. Cariporide (HOE642) has been used in human trials but only in a cardiological setting, and although it has been repeatedly proposed as an anticancer drug in either brain cancer and/or in many other malignant tumors because of its effect as a selective intracellular acidifier of cancer cells of many different lineages, it has never reached any clinical or even preclinical trials in human oncology [37,49,85,86].

Other membrane-bound ion transporters and related mechanisms besides NHE overexpression/inhibition are of fundamental importance in the acid-base regulation of MG, mainly lactate and pH-related glucose transporters and glycolysis [55], MCTs (monocarboxylate transporters), mainly MCT1 and MCT4, and carbonic anhydrase IX (CAIX) [87]. For an integral review on this subject, see ref. [78]. Furthermore, targeting MCTs and CAIX concomitantly with NHE1 inhibition offers a highly promising and integrated approach to the treatment of MG [88,89]. These concepts are the scientific foundation of the treatments proposed in Table 1.

Inhibiting lactate extrusion in MG has also been hypothesized to increase sensitization to radiotherapy by delivering small-molecule MCTs inhibitors to the tumor bed or to the postsurgical resection area [90]. MCT has also been reported to inhibit the invasiveness of GBM while inducing necrosis under different circumstances [70]. Last but not least, a drug like cisplatin, used in MG and many other tumors, has been found to induce IA in vitro and in vivo, a feature that explains whether or not an individual patient responds to treatment with this drug, and perhaps to many others as well [28]. 

Some ion channels are significantly involved in both the regulation of pHi/pHe in cancer cells and in the acquisition of their proliferative and pro-invasive capacities. Ion channels regulate several cell processes, such as cell proliferation, resistance to apoptosis, cell adhesion, cancer cell motility and extracellular matrix invasion. Consequently, an altered physiology of ion channels has also been proposed as a new hallmark of cancer cells and as a potential target for selective therapeutics, either in glioma or in other malignancies [91,92,93,94,95,96,97].

Persistent NHE1 activity in glioma cells is also consistent with depolarized membrane potentials, calcium loading, high pHi and increased cellular Na^+^ levels. While inhibition of NHE1 by cariporide alone seems not to be toxic to glioma cells, its combination with the inhibition of the Na^+^/Ca^2+^ exchanger NCX1.1 selectively kills brain tumor cells [98]. This is consistent with the growing evidence that Ca^2+^ homeostasis is importantly remodeled through the involvement of multiple Ca^2+^ channels and transporters [97]. These molecular mechanisms, that take place at the plasma membrane or in intracellular compartments, participate in enhanced proliferation, cancer cell survival and invasion. As a retro-feedback, intracellular Ca^2+^ concentrations also modulate the activity of NHE1 [99]. Finally, the inhibition of voltage-gated sodium channels, either on their own or through interaction with NHE1, has been proposed in order to prevent cancer growth and progression as well as the metastatic process in different experimental conditions and contexts [13,100].

### 2.2. General Principles of Low pHi-Dependent Cancer Cell Apoptosis as Applied to the Clinical Treatment of Malignant Gliomas 

From the point of view of metabolism, the therapeutic strategy to be recommended in human cancer is mainly directed to achieve an apoptosis-inducing IA of malignant cells while sparing normal cells. This IA must achieve a pHi low enough to induce a chain reaction that leads to selective apoptosis [13,21,43,101,102]. During this apoptotic process the endonuclease DNAase I becomes another fundamental mediating factor [103]. Similarly, a low pHi-induced apoptosis is also mediated through other different mechanisms [21], while the Bcl-2 anti-apoptotic protein seems unable to inhibit it [104]. This is why IA is the main and fundamental weapon of the pH-centered anticancer treatment [102]. IA can be achieved through inhibition of NHE1 and/or H^+^-ATPase and/or CAs IX and XII and/or successful chemotherapy. Most recently, a full issue has been published on the importance of CAs in cancer and other non-oncological pathologies [76].

In all these cases, cells will go into an acid-mediated metabolic collapse and catastrophe which is followed by apoptosis or necrosis [32,105,106]. Other mechanisms of successful cancer treatment include overcoming blockades to cancer cell apoptosis by inhibiting the Bcl-2 anti-apoptotic family, whose activity is, not surprisingly, mediated by intracellular (ic) alkalinization (IA) and inhibited by intracellular (ic)acidification (IAc) [104,107,108,109,110,111,112]. Furthermore, it has been known for a number of years that superoxide (SO) formation, as induced by hormonal or growth factor deprivation, also induces apoptosis of brain cells [113,114], a phenomenon that is further increased after reacting SO with nitric oxide (NO) to form peroxynitrite [115,116]. In contrast, low or physiological concentrations of NO prevent apoptosis [117]. In summary, the different killing mechanisms in cancer cells all share one single and key feature: that they induce a degree of ic acidification incompatible with cellular life. This also contributes to reverse the cancer-specific and pathological hydrogen ion (H^+^) abnormal dynamics of cancer cells and tissues.

#### 2.2.1. An Integrated Approach to Treatment

**Figure 2:**
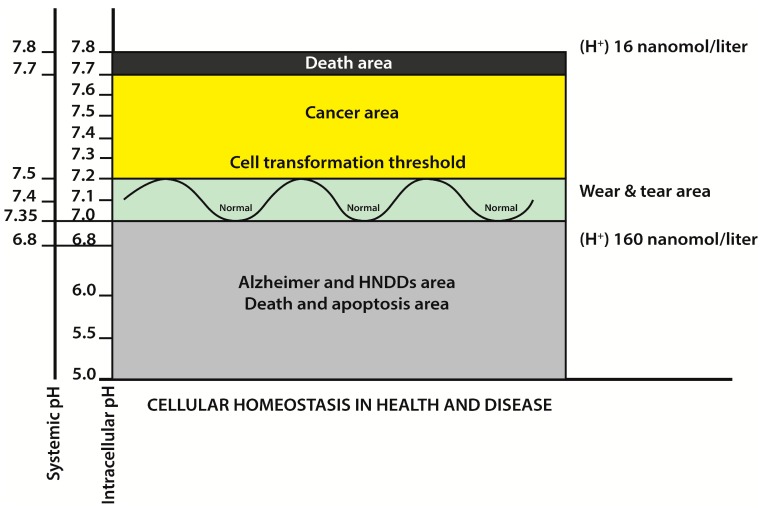
The main aim of the pH-centered treatment is to induce a selective low pHi-mediated apoptosis of all cancer cells. This figure also shows the opposite pH ranges in cancer and in human neurodegenerative diseases (HNNDs). The higher the cellular pH, the lower the hydrogen ion concentrations, and vice versa. For further details, see [13,21].

In spite of all the intratumoral and microenvironmental odds, GBM cells manage to maintain a normal to alkaline intracellular acid-base status in order to protect themselves from a threatening pro-apoptotic IAc. This represents an important part of what we initially called “the neostrategy of cancer cells and tissues” (Figure 2) [13,22,118,119].

IA may be useful as a diagnostic tool, since the potent NHE inhibitor cariporide (HOE642) helps to localize GBM by inducing IA of brain tumors in mice [86]. In the same line, the determination of the effects of different anticancer drugs on pHi is a new and most promising area of research, not only in therapeutics but also in the diagnosis and degree of extension of MG, at least in GBM. This new pH-centered tool allows us to test, even in individual tumors, the sensitivity to IA of different pH-sensitive anticancer drugs such as topiramate, lonidamide, quercetin, dichloroacetate (DCA) and cariporide [30,36,120,121,122,123,124,125,126]. Finally, increasingly sophisticated methodologies to determine pHi and pHe in malignant tumors are being published, while recent and complete reviews on the subject are also available [127].

#### 2.2.2. Intracellular Acidifiers and Anti pH-Related Drugs with Potential Activity in the Treatment of Malignant Gliomas

One of the reasons why most MG are currently incurable is the very high and rapid invasiveness they show, at least in the case of GBM. Their extracellular acidity (EA) is a basic requirement for their highly invasive capacity [127]. Importantly, a two-edged or even triple-edged treatment that addresses simultaneously glucose availability and the pH alterations found in brain tumors may represent an important step forward towards decreasing the migration/invasion process of GBM [102].

Table 2 shows the intracellular acidifiers that through different pHi-lowering mechanisms, mostly related to inhibitory effects on one or more proton extruders (NHE1, CAs, MCTs, H^+^-vacuolar ATPases, etc.), or as voltage gated sodium channels (VGSC) inhibitors, have shown activity against gliomas and promise a potential benefit in the treatment of MG and/or GBM. However, it is most likely that the utilization of only one of them would not be sufficient to regress or even control brain cancer. Thus, a concerted utilization of several of these drugs in pharmacological doses, as has been repeatedly suggested, is mandatory [48,100,128,129]. This should be accompanied by the implementation of cooperative methods that, at least in the case of some of these new and/or repurposed drugs, can overcome the pharmacokinetic blockade imposed by the blood–brain barrier (BBB). To achieve this, the use of polymers, liposomes and nanoparticles has been proposed as a useful method to increase drug delivery to the brain in spite of the BBB [130,131]. In the same line, and in order to increase the efficacy of targeted drug delivery systems with low toxicity, acridine orange, a photodynamic-related acidophilic dye with a strong tumoricidal action in different malignancies, takes advantage of the low extracellular pH of tumors [132,133]. Other photodynamic-oriented procedures have been recently reported as selective and local intracellular acidifiers in cancer treatment [134].

Finally, for a more complete exposure of pH-related acidifiers as anticancer and/or repurposed drugs in preclinical and clinical oncology, see [13,128].

## 3. Conclusions

Glioblastoma multiforme and other malignant brain tumors have a very poor prognosis that shortens both human life and severely worsens its quality in patients with these diseases. The results of standard treatments have been dismal, with minimal and non-significant improvements over recent decades. The treatment of glioblastoma multiforme is, perhaps, the major failure of modern oncological practice. Thus, a different approach is urgently needed to treat this disease. This contribution tries to offer a different approach to that of the orthodox treatments of conventional and mainstream oncological practices in order to implement new therapeutic combinations through schemes that are aimed to reverse the cancer-selective pH-related abnormalities of cancer cells and tissues. For the first time, an integrated and advanced model is considered as a rational approach and alternative proposal for the treatment of malignant brain tumors. Furthermore, the main aim of this pH-centric perspective is to break through the therapeutic impasse and skepticism that dominates this important and disappointing area of modern oncology. The measures proposed here should be tried as an integrated scheme, since its partial utilization would not achieve an efficient glioma cell hyperacidification and selective apoptosis of brain malignancies. This acid-base change, key to the selective energetics of the biochemistry and metabolism of all cancer cells and tissues appears as the most promising target in the treatment of brain cancer. Its full benefits still require further translational research and the activation of clinical trials in bedside neurooncology. We hope that this original perspective may make a difference in the treatment of malignant gliomas on a short-term basis.

## Figures and Tables

**Table 1:** This Table (modified and updated from ref [49]) shows some carcinogenic factors that increase cellular pH through up-regulation of NHE activity.

Carcinogenic Factors That Increase Cellular pH Through Up-Regulation of NHE Activity
Proton transporters-extruders (PTs) and proton pumps (PPs)
Virus (HPV E5 virus: human papiloma virus)
Oncogenes and viral proteins (v-mos, Ha-Ras, HPV16 E7))
Gene products (Bcl-2)
p53 deficiency
Genetic instability and mutations (BRCA1 and BRCA2?)
Chemical carcinogens (benzo(a)pyrene, polycyclic aromatic hydrocarbons, arsenic salts in groundwaters
Chronic hypoxia and HIF
Different mitogens
Hormones and cytokines (Insulin, Growth Hormone, Prolactin, Glucocorticoids, IGF-1, EGF, VEGF, PDGF, Il-1, Il-8, GCSF, TGFß, Angiotensin II, PGE2, Bombesin, Diferric transferrin
Glucose overload
Ageing (“Time causes cancer”- Otto Warburg)

**Table 2:** Drugs with a potential benefit in the treatment of malignant brain tumors that, to a large extent, have not yet been clinically or even preclinically tested.

Drug	Dose and Side Effects	Objective
Topiramate	Starting dose, 50 mg twice a day. The dose must be increased 50 mg every week until reaching 200 mg twice a day.	Topiramate is a voltage gated sodium channel inhibitor that acidifies glioma cells and reduces the risk of seizures [120].
Acetazolamide (AZM)	Starting dose, 125 mg twice a day the first week. And 250 mg twice a day after the first week.	Acetazolamide is a carbonic anhydrase (CA) pan-inhibitor and cell acidifier [76,77].
Amiloride (and/ or liposomal amiloride)	10–30 mg three times a day. Hyperkaliemia can be an occasional problem, more with non-liposomal amiloride.	Amiloride is a non-specific NHE inhibitor and the first one that was developed and introduced in the clinic as a K^+^ sparing diuretic [135]. A positive clinical experience in an occasional patient has been reported [136,137]. Non-liposomal amiloride barely crosses the blood–brain barrier (BBB).
Quercetin	There is no established dose for quercetin. Oral doses of 3 g three times a day are well tolerated in the long term. Very poor oral absorption.	Quercetin is a flavonoid sold over the counter as a nutraceutical, a pan-monocarboxylate transporter (MCT) inhibitor and intracelllular MG acidifier [123,125,138]. Liposomal quercetin is also available.
Fenofibrate	100 mg twice a day.	Fenofibrate is a PPRα agonist that reduces the motility of glioma cells [139], induces their apoptosis [140,141,142,143,144], inhibits glycolytic metabolism [145] and reduces migration [139,146,147]. Fenofibrate also targets glioma stem cells [148,149]. For a further review on fenofibrate see [150], and for a review on fenofibrate in glioma, see [151].
Celecoxib	400 mg twice a day.	Celecoxib inhibits growth and induces apoptosis [152,153,154,155,156,157,158,159,160,161,162]. It also increases the effectiveness of chemotherapeutic drugs [163,164,165,166,167,168,169,170,171] and radiotherapy [172,173,174,175,176]. It attenuates de Wnt/βcatenin pathway [177], reduces angiogenesis [178,179,180,181] and inhibits myeloid derived suppressor cells [182]. For a review on celecoxib in glioma, see [183] and [184].
Cariporide (HOE642) (Unavailable in oncology)		Cariporide (HOE 642) is a powerful NHE1 inhibitor but, unfortunately, is not available for clinical use in oncology. It is orally bioavailable [49]. It also induces non-apoptotic cell death in malignant glioma [98].
Diclofenac (Usual doses)		Diclofenac inhibits lactate formation and counteracts immune suppression in a murine glioma [75].
Dichloroacetate (DCA)	25–40 mg/kg daily in 2–3 weeks cycles (plus Vitamin B1).	DCA is orally available and has been used frequently for GBM in the experimental context as a cell acidifier and glycolytic inhibitor [185,186,187,188], as well as in phase I clinical trials [189,190,191].
Betulinic acid (clinical trials are underway)	Different dosages.	It penetrates the BBB and is highly effective in temozolomide-resistant glioblastoma cells [192,193]. It is also effective against other tumors, like melanoma and neuroectodermic tumors. Its antitumoral activity is also related to its effect as a topoisomerase I inhibitor [194].
Cisplatin (CDDP) (Usual dosages)		Cisplatin induces pHi acidification and a metabolic shift from glycolysis to oxidative metabolism in cervical cancer cells. This is accompanied by the inhibition of cancer cell growth. Cells either recover, maintaining an alkaline pHi to survive and proliferate, although at reduced growth rates, or undergo cell death [28]. CDDP also induces a therapeutic intracellular acidification [29,30,126].
Compound 9t (C9t) (Unavailable)		C9t has been reported to be 500-fold more potent against NHE1 than cariporide and to have a greater selectivity for NHE1 over NHE2 (1400-fold). Besides, C9t is orally bioavailable, has low side-effects in mice and shows a significantly improved safety profile over other NHE1 inhibitors [195].

## References

[1] Calvo F.A., Pastor M.A., Dy C., Alegria E., Anton Aparicio L.M., Gil A., Harguindey S., Zubieta J.L., Martinez Lage M. (1985). Intra-arterial and intravenous chemotherapy for the treatment of malignant glioma. Preliminary results. Am. J. Clin. Oncol..

[2] Harguindey S.S., Kolbeck R.C., Bransome E.D. (1975). Letter: Ureterosigmoidostomy and cancer: New observations. Ann. Intern. Med..

[3] Harguindey S. (1982). Hydrogen ion dynamics and cancer: An appraisal. Med. Pediatr. Oncol..

[4] Lagadic-Gossmann D., Hardonniere K., Mograbi B., Sergent O., Huc L. (2019). Disturbances in H^+^ dynamics during environmental carcinogenesis. Biochimie.

[5] Krex D., Klink B., Hartmann C., von Deimling A., Pietsch T., Simon M., Sabel M., Steinbach J.P., Heese O., Reifenberger G. (2007). Long-term survival with glioblastoma multiforme. Brain.

[6] Limentani S.A., Asher A., Heafner M., Kim J.W., Fraser R. (2005). A phase I trial of surgery, gliadel wafer implantation, and immediate postoperative carboplatin in combination with radiation therapy for primary anaplastic astrocytoma or glioblastoma multiforme. J. Neurooncol..

[7] Shchors K., Massaras A., Hanahan D. (2015). Dual targeting of the autophagic regulatory circuitry in gliomas with repurposed drugs elicits cell-lethal autophagy and therapeutic benefit. Cancer Cell.

[8] Tamiya T., Takao S., Ichikawa T., Chayama K., Date I. (2006). Successful chemotherapy for congenital malignant gliomas: A report of two cases. Pediatr. Neurosurg..

[9] Newton H.B. (2005). Intra-arterial chemotherapy of primary brain tumors. Curr. Treat Options Oncol..

[10] Kast R.E., Boockvar J.A., Bruning A., Cappello F., Chang W.W., Cvek B., Dou Q.P., Duenas-Gonzalez A., Efferth T., Focosi D. (2013). A conceptually new treatment approach for relapsed glioblastoma: Coordinated undermining of survival paths with nine repurposed drugs (CUSP9) by the international initiative for accelerated improvement of glioblastoma care. Oncotarget.

[11] Purow B. (2016). Repurposing existing agents as adjunct therapies for glioblastoma. Neurooncol. Pract..

[12] Harguindey S., Reshkin S.J. (2017). The new ph-centric anticancer paradigm in oncology and medicine. Semin. Cancer Biol..

[13] Harguindey S., Stanciu D., Devesa J., Alfarouk K., Cardone R.A., Polo Orozco J.D., Devesa P., Rauch C., Orive G., Anitua E. (2017). Cellular acidification as a new approach to cancer treatment and to the understanding and therapeutics of neurodegenerative diseases. Semin. Cancer Biol..

[14] Cichocka M., Kozub J., Urbanik A. (2015). pH measurements of the brain using phosphorus magnetic resonance spectroscopy ((31)PMRS) in healthy men - comparison of two analysis methods. Pol. J. Radiol..

[15] Ferrauto G., Di Gregorio E., Auboiroux V., Petit M., Berger F., Aime S., Lahrech H. (2018). CEST-MRI for glioma pH quantification in mouse model: Validation by immunohistochemistry. NMR Biomed..

[16] Harris R.J., Cloughesy T.F., Liau L.M., Prins R.M., Antonios J.P., Li D., Yong W.H., Pope W.B., Lai A., Nghiemphu P.L. (2015). pH-weighted molecular imaging of gliomas using amine chemical exchange saturation transfer MRI. Neuro. Oncol..

[17] Loiselle F.B., Casey J.R. (2010). Measurement of intracellular pH. Methods Mol. Biol..

[18] Kotyk A., Slavík J. (1989). Intracellular pH and Its Measurement.

[19] Lim H., Albatany M., Martinez-Santiesteban F., Bartha R., Scholl T.J. (2018). Longitudinal measurements of intra- and extracellular ph gradient in a rat model of glioma. Tomography.

[20] Obara M., Szeliga M., Albrecht J. (2008). Regulation of pH in the mammalian central nervous system under normal and pathological conditions: Facts and hypotheses. Neurochem. Int..

[21] Harguindey S., Orive G., Cacabelos R., Hevia E.M., de Otazu R.D., Arranz J.L., Anitua E. (2008). An integral approach to the etiopathogenesis of human neurodegenerative diseases (HNDDs) and cancer. Possible therapeutic consequences within the frame of the trophic factor withdrawal syndrome (TFWS). Neuropsychiatr. Dis. Treat..

[22] Honasoge A., Sontheimer H. (2013). Involvement of tumor acidification in brain cancer pathophysiology. Front. Physiol..

[23] Omran Z., Scaife P., Stewart S., Rauch C. (2017). Physical and biological characteristics of multi drug resistance (MDR): An integral approach considering ph and drug resistance in cancer. Semin. Cancer Biol..

[24] Di Cristofori A., Ferrero S., Bertolini I., Gaudioso G., Russo M.V., Berno V., Vanini M., Locatelli M., Zavanone M., Rampini P. (2015). The vacuolar H^+^ ATPase is a novel therapeutic target for glioblastoma. Oncotarget.

[25] Spugnini E.P., Sonveaux P., Stock C., Perez-Sayans M., De Milito A., Avnet S., Garcia A.G., Harguindey S., Fais S. (2015). Proton channels and exchangers in cancer. Biochim. Biophys. Acta..

[26] Perek N., Denoyer D., Dubois F., Koumanov F. (2002). Malignant gliomas display altered plasma membrane potential and ph regulation--interaction with Tc-99m-MIBI and Tc-99m-Tetrofosmin uptakes. Gen. Physiol. Biophys..

[27] Luciani F., Spada M., De Milito A., Molinari A., Rivoltini L., Montinaro A., Marra M., Lugini L., Logozzi M., Lozupone F. (2004). Effect of proton pump inhibitor pretreatment on resistance of solid tumors to cytotoxic drugs. J. Natl. Cancer Inst..

[28] Shirmanova M.V., Druzhkova I.N., Lukina M.M., Dudenkova V.V., Ignatova N.I., Snopova L.B., Shcheslavskiy V.I., Belousov V.V., Zagaynova E.V. (2017). Chemotherapy with cisplatin: Insights into intracellular pH and metabolic landscape of cancer cells in vitro and in vivo. Sci. Rep..

[29] Raudenska M., Balvan J., Fojtu M., Gumulec J., Masarik M. (2019). Unexpected therapeutic effects of cisplatin. Metallomics.

[30] Tamtaji O.R., Mirzaei H., Shamshirian A., Shamshirian D., Behnam M., Asemi Z. (2019). New trends in glioma cancer therapy: Targetig Na^+^/H^+^ exchangers. J. Cell. Physiol..

[31] Pouyssegur J., Sardet C., Franchi A., L’Allemain G., Paris S. (1984). A specific mutation abolishing Na^+^/H^+^antiport activity in hamster fibroblasts precludes growth at neutral and acidic pH. Proc. Natl. Acad. Sci. USA.

[32] Harguindey S., Orive G., Luis Pedraz J., Paradiso A., Reshkin S.J. (2005). The role of pH dynamics and the Na^+^/H^+^ antiporter in the etiopathogenesis and treatment of cancer. Two faces of the same coin--one single nature. Biochim. Biophys. Acta..

[33] Guan X., Luo L., Begum G., Kohanbash G., Song Q., Rao A., Amankulor N., Sun B., Sun D., Jia W. (2018). Elevated Na/H exchanger 1 (SLC9a1) emerges as a marker for tumorigenesis and prognosis in gliomas. J. Exp. Clin. Cancer Res..

[34] Cong D., Zhu W., Shi Y., Pointer K.B., Clark P.A., Shen H., Kuo J.S., Hu S., Sun D. (2014). Upregulation of NHE1 protein expression enables glioblastoma cells to escape tmz-mediated toxicity via increased H^+^ extrusion, cell migration and survival. Carcinogenesis.

[35] McLean L.A., Roscoe J., Jorgensen N.K., Gorin F.A., Cala P.M. (2000). Malignant gliomas display altered pH regulation by nhe1 compared with nontransformed astrocytes. Am. J. Physiol. Cell Physiol..

[36] Flogel U., Willker W., Leibfritz D. (1994). Regulation of intracellular pH in neuronal and glial tumour cells, studied by multinuclear nmr spectroscopy. NMR. Biomed..

[37] Zhu W., Carney K.E., Pigott V.M., Falgoust L.M., Clark P.A., Kuo J.S., Sun D. (2016). Glioma-mediated microglial activation promotes glioma proliferation and migration: Roles of Na^+^/H^+^ exchanger isoform 1. Carcinogenesis.

[38] Huber V., Camisaschi C., Berzi A., Ferro S., Lugini L., Triulzi T., Tuccitto A., Tagliabue E., Castelli C., Rivoltini L. (2017). Cancer acidity: An ultimate frontier of tumor immune escape and a novel target of immunomodulation. Semin. Cancer Biol..

[39] Lacroix R., Rozeman E.A., Kreutz M., Renner K., Blank C.U. (2018). Targeting tumor-associated acidity in cancer immunotherapy. Cancer Immunol. Immunother..

[40] Pillai S.R., Damaghi M., Marunaka Y., Spugnini E.P., Fais S., Gillies R.J. (2019). Causes, consequences, and therapy of tumors acidosis. Cancer Metastasis. Rev..

[41] Pilon-Thomas S., Kodumudi K.N., El-Kenawi A.E., Russell S., Weber A.M., Luddy K., Damaghi M., Wojtkowiak J.W., Mule J.J., Ibrahim-Hashim A. (2016). Neutralization of tumor acidity improves antitumor responses to immunotherapy. Cancer Res..

[42] Wu H., Estrella V., Enriquez-Navas P., El-Kenawi A., Russell S., Abrahams D., Ibrahim-Hashim A., Longo D., Reshetnyak Y., Luddy K. (2019). Lymph nodes inhibit T-cell effector functions locally by establishing acidic niches. BioRxiv.

[43] Rich I.N., Worthington-White D., Garden O.A., Musk P. (2000). Apoptosis of leukemic cells accompanies reduction in intracellular pH after targeted inhibition of the Na^+^/H^+^ exchanger. Blood.

[44] Nijhout H.F. (1990). Metaphors and the role of genes in development. BioEssays.

[45] Thews O., Riemann A. (2019). Tumor pH and metastasis: A malignant process beyond hypoxia. Cancer Metastasis Rev..

[46] Reshkin S.J., Cardone R.A., Harguindey S. (2013). Na^+^-H^+^ exchanger, pH regulation and cancer. Recent Pat. Anti-cancer Drug Discov..

[47] Cardone R.A., Casavola V., Reshkin S.J. (2005). The role of disturbed pH dynamics and the Na^+^/H^+^ exchanger in metastasis. Nat. Rev. Cancer.

[48] Harguindey S., Arranz J.L., Wahl M.L., Orive G., Reshkin S.J. (2009). Proton transport inhibitors as potentially selective anticancer drugs. Anticancer. Res..

[49] Harguindey S., Arranz J.L., Polo Orozco J.D., Rauch C., Fais S., Cardone R.A., Reshkin S.J. (2013). Cariporide and other new and powerful NHE1 inhibitors as potentially selective anticancer drugs--an integral molecular/biochemical/metabolic/clinical approach after one hundred years of cancer research. J. Transl. Med..

[50] Devesa J., Devesa P., Reimunde P. (2010). Growth hormone revisited. Med. Clin. (Barc).

[51] Warburg O. (1956). On the origin of cancer cells. Science.

[52] Warburg OH. The Prime Cause and Prevention of Cancer. Proceedings of the Lecture at the meeting of Nobel Laureates.

[53] Nagata H., Che X.F., Miyazawa K., Tomoda A., Konishi M., Ubukata H., Tabuchi T. (2011). Rapid decrease of intracellular pH associated with inhibition of Na+/H+ exchanger precedes apoptotic events in the MNK45 and MNK74 gastric cancer cell lines treated with 2-aminophenoxazine-3-one. Oncol. Rep..

[54] Quach C.H.T., Jung K.-H., Lee J.H., Park J.W., Moon S.H., Cho Y.S., Choe Y.S., Lee K.-H. (2016). Mild alkalization acutely triggers the Warburg effect by enhancing hexokinase activity via voltage-dependent anion channel binding. PLoS ONE.

[55] Alfarouk K.O., Verduzco D., Rauch C., Muddathir A.K., Adil H.H., Elhassan G.O., Ibrahim M.E., David Polo Orozco J., Cardone R.A., Reshkin S.J. (2014). Glycolysis, tumor metabolism, cancer growth and dissemination. A new pH-based etiopathogenic perspective and therapeutic approach to an old cancer question. Oncoscience.

[56] Hardonniere K., Huc L., Sergent O., Holme J.A., Lagadic-Gossmann D. (2017). Environmental carcinogenesis and pH homeostasis: Not only a matter of dysregulated metabolism. Semin. Cancer Biol..

[57] Aravena C., Beltran A.R., Cornejo M., Torres V., Diaz E.S., Guzman-Gutierrez E., Pardo F., Leiva A., Sobrevia L., Ramirez M.A. (2012). Potential role of sodium-proton exchangers in the low concentration arsenic trioxide-increased intracellular pH and cell proliferation. PLoS ONE.

[58] Orive G., Reshkin S.J., Harguindey S., Pedraz J.L. (2003). Hydrogen ion dynamics and the Na^+^/H^+^ exchanger in cancer angiogenesis and antiangiogenesis. Br. J. Cancer.

[59] Devesa J., Alonso A., Lopez N., Garcia J., Puell C.I., Pablos T., Devesa P. (2017). Growth hormone (GH) and rehabilitation promoted distal innervation in a child affected by caudal regression syndrome. Int. J. Mol. Sci..

[60] Devesa J., Nunez I., Agra C., Bejarano A., Devesa P. (2018). Treatment with growth hormone (GH) increased the metabolic activity of the brain in an elder patient, not gh-deficient, who suffered mild cognitive alterations and had an apoe 4/3 genotype. Int. J. Mol. Sci..

[61] Hartman M.L., Xu R., Crowe B.J., Robison L.L., Erfurth E.M., Kleinberg D.L., Zimmermann A.G., Woodmansee W.W., Cutler G.B., Chipman J.J. (2013). Prospective safety surveillance of gh-deficient adults: Comparison of gh-treated vs. untreated patients. J. Clin. Endocrinol. Metab..

[62] Indini A., Schiavello E., Biassoni V., Bergamaschi L., Magni M.C., Puma N., Chiaravalli S., Pallotti F., Seregni E., Diletto B. (2017). Long-term safety of growth hormone replacement therapy after childhood medulloblastoma and PNET: It is time to set aside old concerns. J. Neuro-Oncol..

[63] Perry J.K., Mohankumar K.M., Emerald B.S., Mertani H.C., Lobie P.E. (2008). The contribution of growth hormone to mammary neoplasia. J. Mammary Gland. Biol. Neoplasia..

[64] Perry J.K., Emerald B.S., Mertani H.C., Lobie P.E. (2006). The oncogenic potential of growth hormone. Growth Horm IGF. Res..

[65] Chhabra Y., Waters M.J., Brooks A.J. (2011). Role of the growth hormone-IGF-1 axis in cancer. Expert Rev. Endocrinol. Metab..

[66] Lea R.W., Dawson T., Martinez-Moreno C.G., El-Abry N., Harvey S. (2015). Growth hormone and cancer: GH production and action in glioma?. Gen. Comp. Endocrinol..

[67] Moolenaar W.H. (1986). Effects of growth factors on intracellular pH regulation. Annu. Rev. Physiol..

[68] L’Allemain G., Paris S., Pouyssegur J. (1984). Growth factor action and intracellular ph regulation in fibroblasts. Evidence for a major role of the Na^+^/H^+^ antiport. J. Biol. Chem..

[69] He B., Deng C., Zhang M., Zou D., Xu M. (2007). Reduction of intracellular pH inhibits the expression of vegf in K562 cells after targeted inhibition of the Na^+^/H^+^ exchanger. Leuk. Res..

[70] Colen C.B., Shen Y., Ghoddoussi F., Yu P., Francis T.B., Koch B.J., Monterey M.D., Galloway M.P., Sloan A.E., Mathupala S.P. (2011). Metabolic targeting of lactate efflux by malignant glioma inhibits invasiveness and induces necrosis: An in vivo study. Neoplasia.

[71] Geeviman K., Babu D., Prakash Babu P. (2018). Pantoprazole induces mitochondrial apoptosis and attenuates NFf-KAPPAB signaling in glioma cells. Cell Mol. Neurobiol..

[72] Berrino E., Supuran C.T. (2019). Novel approaches for designing drugs that interfere with pH regulation. Expert Opin. Drug Discov..

[73] Peretti M., Raciti F.M., Carlini V., Verduci I., Sertic S., Barozzi S., Garre M., Pattarozzi A., Daga A., Barbieri F. (2018). Mutual influence of ROS, pH, and CLIC1 membrane protein in the regulation of G1-S phase progression in human glioblastoma stem cells. Mol. Cancer Ther..

[74] Lee S.H., McIntyre D., Honess D., Hulikova A., Pacheco-Torres J., Cerdan S., Swietach P., Harris A.L., Griffiths J.R. (2018). Carbonic anhydrase IX is a pH-stat that sets an acidic tumour extracellular pH in vivo. Br. J. Cancer.

[75] Chirasani S.R., Leukel P., Gottfried E., Hochrein J., Stadler K., Neumann B., Oefner P.J., Gronwald W., Bogdahn U., Hau P. (2013). Diclofenac inhibits lactate formation and efficiently counteracts local immune suppression in a murine glioma model. Int. J. Cancer.

[76] Supuran C.T. (2018). Carbonic anhydrases and metabolism. Metabolites.

[77] Mboge M.Y., Mahon B.P., McKenna R., Frost S.C. (2018). Carbonic anhydrases: Role in pH control and cancer. Metabolites.

[78] Miranda-Goncalves V., Reis R.M., Baltazar F. (2016). Lactate transporters and pH regulation: Potential therapeutic targets in glioblastomas. Curr. Cancer Drug Targets.

[79] Sontheimer H. (2004). Ion channels and amino acid transporters support the growth and invasion of primary brain tumors. Mol. Neurobiol..

[80] Sontheimer H. (2008). An unexpected role for ion channels in brain tumor metastasis. Exp. Biol. Med. (Maywood).

[81] Cong D., Zhu W., Kuo J.S., Hu S., Sun D. (2015). Ion transporters in brain tumors. Curr. Med. Chem..

[82] Kroemer G., Pouyssegur J. (2008). Tumor cell metabolism: Cancer’s achilles’ heel. Cancer Cell.

[83] Parks S.K., Pouyssegur J. (2017). Targeting pH regulating proteins for cancer therapy-progress and limitations. Semin. Cancer Biol..

[84] Guan X., Hasan M.N., Begum G., Kohanbash G., Carney K.E., Pigott V.M., Persson A.I., Castro M.G., Jia W., Sun D. (2018). Blockade of Na/H exchanger stimulates glioma tumor immunogenicity and enhances combinatorial tmz and anti-pd-1 therapy. Cell Death Dis..

[85] Harguindey S., Koltai T., Reshkin S.J. (2018). Curing cancer? Further along the new pH-centric road and paradigm. Oncoscience.

[86] Albatany M., Li A., Meakin S., Bartha R. (2018). In vivo detection of acute intracellular acidification in glioblastoma multiforme following a single dose of cariporide. Int. J. Clin. Oncol..

[87] Aggarwal M., Kondeti B., McKenna R. (2013). Anticonvulsant/antiepileptic carbonic anhydrase inhibitors: A patent review. Expert Opin. Ther. Pat..

[88] Perez-Escuredo J., Van Hee V.F., Sboarina M., Falces J., Payen V.L., Pellerin L., Sonveaux P. (2016). Monocarboxylate transporters in the brain and in cancer. Biochim. Biophys. Acta..

[89] Miranda-Goncalves V., Honavar M., Pinheiro C., Martinho O., Pires M.M., Pinheiro C., Cordeiro M., Bebiano G., Costa P., Palmeirim I. (2013). Monocarboxylate transporters (MCTs) in gliomas: Expression and exploitation as therapeutic targets. Neuro. Oncol..

[90] Colen C.B., Seraji-Bozorgzad N., Marples B., Galloway M.P., Sloan A.E., Mathupala S.P. (2006). Metabolic remodeling of malignant gliomas for enhanced sensitization during radiotherapy: An in vitro study. Neurosurgery.

[91] Schwab A., Stock C. (2014). Ion channels and transporters in tumour cell migration and invasion. Philosophical transactions of the Royal Society of London. Philos. Trans. R. Soc. Lond. B Biol. Sci..

[92] Litan A., Langhans S.A. (2015). Cancer as a channelopathy: Ion channels and pumps in tumor development and progression. Front. Cell. Neurosci..

[93] Lang F., Stournaras C. (2014). Ion channels in cancer: Future perspectives and clinical potential. Philos. Trans. R. Soc. Lond. B Biol. Sci..

[94] Besson P., Driffort V., Bon E., Gradek F., Chevalier S., Roger S. (2015). How do voltage-gated sodium channels enhance migration and invasiveness in cancer cells?. Biochim. Biophys. Acta.

[95] Roger S., Gillet L., Le Guennec J.Y., Besson P. (2015). Voltage-gated sodium channels and cancer: Is excitability their primary role?. Front. Pharmacol..

[96] Stock C., Ludwig F.T., Hanley P.J., Schwab A. (2013). Roles of ion transport in control of cell motility. Compr. Physiol..

[97] Prevarskaya N., Ouadid-Ahidouch H., Skryma R., Shuba Y. (2014). Remodelling of Ca2+ transport in cancer: How it contributes to cancer hallmarks?. Philos. Trans. R. Soc. Lond. B Biol. Sci..

[98] Harley W., Floyd C., Dunn T., Zhang X.D., Chen T.Y., Hegde M., Palandoken H., Nantz M.H., Leon L., Carraway K.L. (2010). Dual inhibition of sodium-mediated proton and calcium efflux triggers non-apoptotic cell death in malignant gliomas. Brain Res..

[99] Rice J.J., Martino M.M., De Laporte L., Tortelli F., Briquez P.S., Hubbell J.A. (2013). Engineering the regenerative microenvironment with biomaterials. Adv. Healthc. Mater..

[100] Koltai T. (2015). Voltage-gated sodium channel as a target for metastatic risk reduction with re-purposed drugs. F1000Research.

[101] Persi E., Duran-Frigola M., Damaghi M., Roush W.R., Aloy P., Cleveland J.L., Gillies R.J., Ruppin E. (2018). Systems analysis of intracellular ph vulnerabilities for cancer therapy. Nat. Commun..

[102] Koltai T. (2017). Triple-edged therapy targeting intracellular alkalosis and extracellular acidosis in cancer. Semin. Cancer Biol..

[103] Heon J. (1995). Effects of intracellular pH on apoptosis in hl-60 human leukemia cells. Yonsei. Med. J..

[104] Zanke B.W., Lee C., Arab S., Tannock I.F. (1998). Death of tumor cells after intracellular acidification is dependent on stress-activated protein kinases (SAPK/JNK) pathway activation and cannot be inhibited by Bcl-2 expression or interleukin 1beta-converting enzyme inhibition. Cancer Res..

[105] Di Sario A., Bendia E., Omenetti A., De Minicis S., Marzioni M., Kleemann H.W., Candelaresi C., Saccomanno S., Alpini G., Benedetti A. (2007). Selective inhibition of ion transport mechanisms regulating intracellular ph reduces proliferation and induces apoptosis in cholangiocarcinoma cells. Dig. Liver Dis..

[106] Letai A.G. (2008). Diagnosing and exploiting cancer’s addiction to blocks in apoptosis. Nat. Rev. Cancer.

[107] Reynolds J.E., Li J., Craig R.W., Eastman A. (1996). Bcl-2 and MCL-1 expression in chinese hamster ovary cells inhibits intracellular acidification and apoptosis induced by staurosporine. Exp. Cell Res..

[108] Xie Z., Schendel S., Matsuyama S., Reed J.C. (1998). Acidic pH promotes dimerization of Bcl-2 family proteins. Biochemistry.

[109] Thangaraju M., Sharma K., Leber B., Andrews D.W., Shen S.H., Srikant C.B. (1999). Regulation of acidification and apoptosis by SHP-1 and Bcl-2. J. Biol. Chem..

[110] Takahashi A., Masuda A., Sun M., Centonze V.E., Herman B. (2004). Oxidative stress-induced apoptosis is associated with alterations in mitochondrial caspase activity and Bcl-2-dependent alterations in mitochondrial pH (pHm). Brain Res. Bull..

[111] Roepe P.D. (2001). Ph and multidrug resistance. Novartis. Found Symp..

[112] Harguindey S., Pedraz J.L., Canero R.G., Katin M. (2000). Edelfosine, apoptosis, MDR and the Na^+^/H^+^ exchanger: Induction mechanisms and treatment implications. Apoptosis.

[113] Lieberthal W., Triaca V., Koh J.S., Pagano P.J., Levine J.S. (1998). Role of superoxide in apoptosis induced by growth factor withdrawal. Am. J. Physiol..

[114] Harguindey S., Reshkin S.J., Orive G., Arranz J.L., Anitua E. (2007). Growth and trophic factors, pH and the Na^+^/H^+^ exchanger in alzheimer’s disease, other neurodegenerative diseases and cancer: New therapeutic possibilities and potential dangers. Curr. Alzheimer. Res..

[115] Estevez A.G., Jordan J. (2002). Nitric oxide and superoxide, a deadly cocktail. Ann. N. Y. Acad. Sci..

[116] Espey M.G., Miranda K.M., Thomas D.D., Xavier S., Citrin D., Vitek M.P., Wink D.A. (2002). A chemical perspective on the interplay between no, reactive oxygen species, and reactive nitrogen oxide species. Ann. N. Y. Acad. Sci..

[117] Choi B.M., Pae H.O., Jang S.I., Kim Y.M., Chung H.T. (2002). Nitric oxide as a pro-apoptotic as well as anti-apoptotic modulator. J. Biochem. Mol. Biol..

[118] Wenger K.J., Hattingen E., Franz K., Steinbach J.P., Bahr O., Pilatus U. (2017). Intracellular ph measured by ^31^P-MR-spectroscopy might predict site of progression in recurrent glioblastoma under antiangiogenic therapy. J. Magn. Reson. Imaging..

[119] Reshkin S.J., Bellizzi A., Caldeira S., Albarani V., Malanchi I., Poignee M., Alunni-Fabbroni M., Casavola V., Tommasino M. (2000). Na^+^/H^+^ exchanger-dependent intracellular alkalinization is an early event in malignant transformation and plays an essential role in the development of subsequent transformation-associated phenotypes. FASEB. J..

[120] Marathe K., McVicar N., Li A., Bellyou M., Meakin S., Bartha R. (2016). Topiramate induces acute intracellular acidification in glioblastoma. J. Neuro-Oncol..

[121] McVicar N., Li A.X., Meakin S.O., Bartha R. (2015). Imaging chemical exchange saturation transfer (CEST) effects following tumor-selective acidification using lonidamine. NMR. Biomed..

[122] Nath K., Nelson D.S., Ho A.M., Lee S.C., Darpolor M.M., Pickup S., Zhou R., Heitjan D.F., Leeper D.B., Glickson J.D. (2013). (31) P and (1) H MRS of DB-1 xenografts: Lonidamine selectively decreases tumor intracellular pH and energy status and sensitizes tumors to melphalan. NMR. Biomed..

[123] Albatany M., Meakin S., Bartha R. (2018). The monocarboxylate transporter inhibitor quercetin induces intracellular acidification in a mouse model of glioblastoma multiforme: In-vivo detection using magnetic resonance imaging. Investig. New Drugs.

[124] Albatany M., Li A., Meakin S., Bartha R. (2018). Dichloroacetate induced intracellular acidification in glioblastoma: In vivo detection using AACID-CEST MRI at 9.4 Tesla. J. Neurooncol..

[125] Srivastava S., Somasagara R.R., Hegde M., Nishana M., Tadi S.K., Srivastava M., Choudhary B., Raghavan S.C. (2016). Quercetin, a natural flavonoid interacts with DNA, arrests cell cycle and causes tumor regression by activating mitochondrial pathway of apoptosis. Sci. Rep..

[126] Albatany M., Ostapchenko V.G., Meakin S., Bartha R. (2019). Brain tumor acidification using drugs simultaneously targeting multiple pH regulatory mechanisms. J. Neurooncol..

[127] Anemone A., Consolino L., Arena F., Capozza M., Longo D.L. (2019). Imaging tumor acidosis: A survey of the available techniques for mapping in vivo tumor pH. Cancer Metastasis Rev..

[128] Koltai T., Harguindey S., Reshkin S.J. (2019). An Innovative Approach to Understanding and Treating Cancer: Targeting pH.

[129] Koltai T. (2016). Cancer: Fundamentals behind pH targeting and the double edged approach. Oncotargets Ther..

[130] Orive G., Anitua E., Pedraz J.L., Emerich D.F. (2009). Biomaterials for promoting brain protection, repair and regeneration. Nat. Rev. Neurosci..

[131] Pérez-Herrero E., Fernández-Medarde A. (2015). Advanced targeted therapies in cancer: Drug nanocarriers, the future of chemotherapy. Eur. J. Pharm. Biopharm..

[132] Kusuzaki K., Matsubara T., Murata H., Logozzi M., Iessi E., Di Raimo R., Carta F., Supuran C.T., Fais S. (2017). Natural extracellular nanovesicles and photodynamic molecules: Is there a future for drug delivery?. J. Enzym. Inhib. Med. Chem..

[133] Iessi E., Logozzi M., Lugini L., Azzarito T., Federici C., Spugnini E.P., Mizzoni D., Di Raimo R., Angelini D.F., Battistini L. (2017). Acridine orange/exosomes increase the delivery and the effectiveness of acridine orange in human melanoma cells: A new prototype for theranostics of tumors. J. Enzym. Inhib. Med. Chem..

[134] Gdovin M.J., Kadri N., Rios L., Holliday S., Jordan Z. (2017). Focal photodynamic intracellular acidification as a cancer therapeutic. Semin. Cancer Biol..

[135] Harguindey S. (1992). Use of Na^+^/H^+^ antiporter inhibitors as a novel approach to cancer treatment. Amiloride and Its Analogs: Unique Cation Transport Inhibitors.

[136] Wolff J.E., Rytting M.E., Vats T.S., Zage P.E., Ater J.L., Woo S., Kuttesch J., Ketonen L., Mahajan A. (2012). Treatment of recurrent diffuse intrinsic pontine glioma: The MD anderson cancer center experience. J. Neurooncol..

[137] Harguindey S., Orive G., Pedraz J.L., Bello G., Arranz J.L., Samaniego J.M. (2002). Apparent cure of a case of metastatic ovarian carcinoma after the chronic treatment with Na^+^/H^+^ antiport inhibitors. Oncologia.

[138] National Toxicology Program (1992). Toxicology and carcinogenesis studies of quercetin (cas nº. 117-39-5) in f344 rats (feed studies). Natl. Toxicol. Program Tech. Rep. Ser..

[139] Drukala J., Urbanska K., Wilk A., Grabacka M., Wybieralska E., Del Valle L., Madeja Z., Reiss K. (2010). ROS accumulation and i IGR-IR inhibition contribute to fenofibrate/pparalpha -mediated inhibition of glioma cell motility in vitro. Mol. Cancer.

[140] Binello E., Mormone E., Emdad L., Kothari H., Germano I.M. (2014). Characterization of fenofibrate-mediated anti-proliferative pro-apoptotic effects on high-grade gliomas and anti-invasive effects on glioma stem cells. J. Neurooncol..

[141] Han D.F., Zhang J.X., Wei W.J., Tao T., Hu Q., Wang Y.Y., Wang X.F., Liu N., You Y.P. (2015). Fenofibrate induces G0/G1 phase arrest by modulating the PPARα/FoxO1/p27 kip pathway in human glioblastoma cells. Tumour. Biol..

[142] Kast R.E., Hill Q.A., Wion D., Mellstedt H., Focosi D., Karpel-Massler G., Heiland T., Halatsch M.E. (2017). Glioblastoma-synthesized G-CSFsf and GM-CSF contribute to growth and immunosuppression: Potential therapeutic benefit from dapsone, fenofibrate, and ribavirin. Tumour. Biol..

[143] Wilk A., Wyczechowska D., Zapata A., Dean M., Mullinax J., Marrero L., Parsons C., Peruzzi F., Culicchia F., Ochoa A. (2015). Molecular mechanisms of fenofibrate-induced metabolic catastrophe and glioblastoma cell death. Mol. Cell. Biol..

[144] Wilk A., Urbanska K., Grabacka M., Mullinax J., Marcinkiewicz C., Impastato D., Estrada J.J., Reiss K. (2012). Fenofibrate-induced nuclear translocation of FoxO3A triggers bim-mediated apoptosis in glioblastoma cells in vitro. Cell Cycle.

[145] Han D., Wei W., Chen X., Zhang Y., Wang Y., Zhang J., Wang X., Yu T., Hu Q., Liu N. (2015). NF-kB/RelA-PKM2 mediates inhibition of glycolysis by fenofibrate in glioblastoma cells. Oncotarget.

[146] Su C., Shi A., Cao G., Tao T., Chen R., Hu Z., Shen Z., Tao H., Cao B., Hu D. (2015). Fenofibrate suppressed proliferation and migration of human neuroblastoma cells via oxidative stress dependent of txnip upregulation. Biochem. Biophys. Res. Commun..

[147] Shi Y., Tao T., Liu N., Luan W., Qian J., Li R., Hu Q., Wei Y., Zhang J., You Y. (2016). PPARα, a predictor of patient survival in glioma, inhibits cell growth through theE2F1/miR-19a feedback loop. Oncotarget.

[148] Binello E., Germano I.M. (2011). Targeting glioma stem cells: A novel framework for brain tumors. Cancer Sci..

[149] Binello E., Germano I.M. (2014). P17.07: Fenofibrate as a novel therapeutic adjuvant for targeting glioma stem cells. Neuro-oncology.

[150] Koltai T. (2015). Fenofibrate in cancer: Mechanisms involved in anticancer activity. F1000Research.

[151] Lian X., Wang G., Zhou H., Zheng Z., Fu Y., Cai L. (2018). Anticancer properties of fenofibrate: A repurposing use. J. Cancer.

[152] Chen J.C., Chen Y., Su Y.H., Tseng S.H. (2007). Celecoxib increased expression of 14-3-3sigma and induced apoptosis of glioma cells. Anticancer Res..

[153] Nam D.H., Park K., Park C., Im Y.H., Kim M.H., Lee S., Hong S.C., Shin H.J., Kim J.H., Eoh W. (2004). Intracranial inhibition of glioma cell growth by cyclooxygenase-2 inhibitor celecoxib. Oncol. Rep..

[154] Kang K.B., Zhu C., Yong S.K., Gao Q., Wong M.C. (2009). Enhanced sensitivity of celecoxib in human glioblastoma cells: Induction of DNA damage leading to p53-dependent g1 cell cycle arrest and autophagy. Mol. Cancer.

[155] Gaiser T., Becker M.R., Habel A., Reuss D.E., Ehemann V., Rami A., Siegelin M.D. (2008). Trail-mediated apoptosis in malignant glioma cells is augmented by celecoxib through proteasomal degradation of survivin. Neurosci. Lett..

[156] Sareddy G.R., Geeviman K., Ramulu C., Babu P.P. (2012). The nonsteroidal anti-inflammatory drug celecoxib suppresses the growth and induces apoptosis of human glioblastoma cells via the nf-kappab pathway. J. Neurooncol..

[157] Zhou R., Zhang L.Z., Wang R.Z. (2010). Effect of celecoxib on proliferation, apoptosis, and survivin expression in human glioma cell line u251. Chin. J. Cancer.

[158] Sharma V., Dixit D., Ghosh S., Sen E. (2011). COX-2 regulates the proliferation of glioma stem like cells. Neurochem. Int..

[159] Yerokun T., Winfield L.L. (2015). Celecoxib and llw-3-6 reduce survival of human glioma cells independently and synergistically with sulfasalazine. Anticancer Res..

[160] Bernardi A., Jacques-Silva M.C., Delgado-Canedo A., Lenz G., Battastini A.M. (2006). Nonsteroidal anti-inflammatory drugs inhibit the growth of C6 and U138-MG glioma cell lines. Eur. J. Pharmacol..

[161] Sato A., Mizobuchi Y., Nakajima K., Shono K., Fujihara T., Kageji T., Kitazato K., Matsuzaki K., Mure H., Kuwayama K. (2017). Blocking COX-2 induces apoptosis and inhibits cell proliferation via the Akt/Survivin- and Akt/ID3 pathway in low-grade-glioma. J. Neurooncol..

[162] Cherukuri D.P., Nelson M.A. (2004). Glioma growth inhibition by selective COX-2 inhibitors via cyclooxygenase independent pathways: Implications for therapy. Cancer Biol. Ther..

[163] Reardon D.A., Quinn J.A., Vredenburgh J., Rich J.N., Gururangan S., Badruddoja M., Herndon J.E., Dowell J.M., Friedman A.H., Friedman H.S. (2005). Phase ii trial of irinotecan plus celecoxib in adults with recurrent malignant glioma. Cancer.

[164] Kang S.G., Kim J.S., Park K., Kim J.S., Groves M.D., Nam D.H. (2006). Combination celecoxib and temozolomide in C6 rat glioma orthotopic model. Oncol. Rep..

[165] Walbert T., Gilbert M.R., Groves M.D., Puduvalli V.K., Yung W.K., Conrad C.A., Bobustuc G.C., Colman H., Hsu S.H., Bekele B.N. (2011). Combination of 6-thioguanine, capecitabine, and celecoxib with temozolomide or lomustine for recurrent high-grade glioma. J. Neurooncol..

[166] Levin V.A., Giglio P., Puduvalli V.K., Jochec J., Groves M.D., Yung W.K., Hess K. (2006). Combination chemotherapy with 13-cis-retinoic acid and celecoxib in the treatment of glioblastoma multiforme. J. Neurooncol..

[167] Stockhammer F., Misch M., Koch A., Czabanka M., Plotkin M., Blechschmidt C., Tuettenberg J., Vajkoczy P. (2010). Continuous low-dose temozolomide and celecoxib in recurrent glioblastoma. J. Neurooncol..

[168] Pannullo S., Balmaceda C., Serventi J. Temozolomide plus celecoxib for treatment of malignant gliomas [abstract]. Proceedings of the 39th ASCO Annual Meeting.

[169] Pannullo S., Burton J., Serventi J., Stieg P., Subramanian H., Elsoueidi R., El-Jassous I., Balmaceda C. (2006). Phase I/II trial of twice-daily temozolomide and celecoxib for treatment of relapsed malignant glioma: Final data. J. Clin. Oncol..

[170] Vera M., Barcia E., Negro S., Marcianes P., Garcia-Garcia L., Slowing K., Fernandez-Carballido A. (2014). New celecoxib multiparticulate systems to improve glioblastoma treatment. Int. J. Pharm..

[171] Welzel G., Gehweiler J., Brehmer S., Appelt J.U., von Deimling A., Seiz-Rosenhagen M., Schmiedek P., Wenz F., Giordano F.A. (2015). Metronomic chemotherapy with daily low-dose temozolomide and celecoxib in elderly patients with newly diagnosed glioblastoma multiforme: A retrospective analysis. J. Neurooncol..

[172] Qin S.B., Zhou L.Y., Xu X.T. (2008). Radiosensitization of celecoxib for glioma cells. J. Oncol..

[173] Ma H.-I., Chiou S.-H., Hueng D.-Y., Tai L.-K., Huang P.-I., Kao C.-L., Chen Y.-W., Sytwu H.-K. (2011). Celecoxib and radioresistant glioblastoma-derived CD133+ cells: Improvement in radiotherapeutic effects. J. Neurooncol..

[174] Chen K.H., Hsu C.C., Song W.S., Huang C.S., Tsai C.C., Kuo C.D., Hsu H.S., Tsai T.H., Tsai C.Y., Woung L.C. (2010). Celecoxib enhances radiosensitivity in medulloblastoma-derived CD133-positive cells. Childs Nerv. Syst..

[175] Kuipers G.K., Slotman B.J., Wedekind L.E., Stoter T.R., Berg J., Sminia P., Lafleur M.V. (2007). Radiosensitization of human glioma cells by cyclooxygenase-2 (COX-2) inhibition: Independent on cox-2 expression and dependent on the cox-2 inhibitor and sequence of administration. Int. J. Radiat. Biol..

[176] Kang K.B., Wang T.T., Woon C.T., Cheah E.S., Moore X.L., Zhu C., Wong M.C. (2007). Enhancement of glioblastoma radioresponse by a selective COX-2 inhibitor celecoxib: Inhibition of tumor angiogenesis with extensive tumor necrosis. Int. J. Radiat. Oncol. Biol. Phys..

[177] Sareddy G.R., Kesanakurti D., Kirti P.B., Babu P.P. (2013). Nonsteroidal anti-inflammatory drugs diclofenac and celecoxib attenuates WNT/beta-catenin/tcf signaling pathway in human glioblastoma cells. Neurochem. Res..

[178] Ju R.J., Zeng F., Liu L., Mu L.M., Xie H.J., Zhao Y., Yan Y., Wu J.S., Hu Y.J., Lu W.L. (2016). Destruction of vasculogenic mimicry channels by targeting epirubicin plus celecoxib liposomes in treatment of brain glioma. Int. J. Nanomedicine..

[179] Porkholm M., Valanne L., Lonnqvist T., Holm S., Lannering B., Riikonen P., Wojcik D., Sehested A., Clausen N., Harila-Saari A. (2014). Radiation therapy and concurrent topotecan followed by maintenance triple anti-angiogenic therapy with thalidomide, etoposide, and celecoxib for pediatric diffuse intrinsic pontine glioma. Pediatr. Blood Cancer.

[180] Virrey J.J., Liu Z., Cho H.Y., Kardosh A., Golden E.B., Louie S.G., Gaffney K.J., Petasis N.A., Schonthal A.H., Chen T.C. (2010). Antiangiogenic activities of 2,5-dimethyl-celecoxib on the tumor vasculature. Mol. Cancer Ther..

[181] Kerschbaumer J., Schmidt F.A., Grams A.E., Nowosielski M., Pinggera D., Brawanski K.R., Petr O., Thome C., Tuettenberg J., Seiz M. (2015). Dual anti-angiogenic chemotherapy with temozolomide and celecoxib in selected patients with malignant glioma not eligible for standard treatment. Anticancer Res..

[182] Fujita M., Kohanbash G., Fellows-Mayle W., Hamilton R.L., Komohara Y., Decker S.A., Ohlfest J.R., Okada H. (2011). COX-2 blockade suppresses gliomagenesis by inhibiting myeloid-derived suppressor cells. Cancer Res..

[183] Giglio P., Levin V. (2004). Cyclooxygenase-2 inhibitors in glioma therapy. Am. J. Ther..

[184] Schonthal A.H. (2010). Exploiting cyclooxygenase-(in)dependent properties of COX-2 inhibitors for malignant glioma therapy. Anticancer Agents Med. Chem..

[185] Anemone A., Consolino L., Conti L., Reineri F., Cavallo F., Aime S., Longo D.L. (2017). In vivo evaluation of tumour acidosis for assessing the early metabolic response and onset of resistance to dichloroacetate by using magnetic resonance ph imaging. Int. J. Oncol..

[186] Duan Y., Zhao X., Ren W., Wang X., Yu K.F., Li D., Zhang X., Zhang Q. (2013). Antitumor activity of dichloroacetate on C6 glioma cell: In vitro and in vivo evaluation. Oncol. Targets Ther..

[187] Kolesnik D., Pyaskovskaya O., Boichuk I., Solyanik G. (2014). Hypoxia enhances antitumor activity of dichloroacetate. Exp. Oncol..

[188] Fedorchuk A.G., Pyaskovskaya O.N., Gorbik G.V., Prokhorova I.V., Kolesnik D.L., Solyanik G.I. (2016). Effectiveness of sodium dichloroacetate against glioma C6 depends on administration schedule and dosage. Exp. Oncol..

[189] Dunbar E.M., Coats B.S., Shroads A.L., Langaee T., Lew A., Forder J.R., Shuster J.J., Wagner D.A., Stacpoole P.W. (2014). Phase 1 trial of dichloroacetate (DCA) in adults with recurrent malignant brain tumors. Investig. New Drugs.

[190] Lorenzo R.J., Jahn S.C. (2015). Dichloroacetate-phase 1 trial in adults with malignant brain tumors. J. Postdr. Res. Febr..

[191] Chu Q.S., Sangha R., Spratlin J., Vos L.J., Mackey J.R., McEwan A.J., Venner P., Michelakis E.D. (2015). A phase 1 open-labeled, single-arm, dose-escalation, study of dichloroacetate (DCA) in patients with advanced solid tumors. Investig. New Drugs.

[192] Lo W.-L., Huang Y.-N., Hsu T.-I., Chuang J.-Y. (2017). Ddis-14. The effect of betulinic acid on temozolomide-resistant glioblastoma cells. Neuro. Oncol..

[193] Wick W., Grimmel C., Wagenknecht B., Dichgans J., Weller M. (1999). Betulinic acid-induced apoptosis in glioma cells: A sequential requirement for new protein synthesis, formation of reactive oxygen species, and caspase processing. J. Pharmacol. Exp. Ther..

[194] Chowdhury A.R., Mandal S., Mittra B., Sharma S., Mukhopadhyay S., Majumder H.K. (2002). Betulinic acid, a potent inhibitor of eukaryotic topoisomerase i: Identification of the inhibitory step, the major functional group responsible and development of more potent derivatives. Med. Sci. Monit..

[195] Atwal K.S., O’Neil S.V., Ahmad S., Doweyko L., Kirby M., Dorso C.R., Chandrasena G., Chen B.C., Zhao R., Zahler R. (2006). Synthesis and biological activity of 5-aryl-4-(4-(5-methyl-1h-imidazol-4-yl)piperidin-1-yl)pyrimidine analogs as potent, highly selective, and orally bioavailable nhe-1 inhibitors. Bioorganic Med. Chem. Lett..

